# Evidence for Directional Selection at a Novel Major Histocompatibility Class I Marker in Wild Common Frogs (*Rana temporaria*) Exposed to a Viral Pathogen (*Ranavirus*)

**DOI:** 10.1371/journal.pone.0004616

**Published:** 2009-02-25

**Authors:** Amber G. F. Teacher, Trenton W. J. Garner, Richard A. Nichols

**Affiliations:** 1 School of Biological and Chemical Sciences, Queen Mary, University of London, London, United Kingdom; 2 Wildlife Epidemiology, Institute of Zoology, Zoological Society of London, Regent's Park, London, United Kingdom; American Museum of Natural History, United States of America

## Abstract

Whilst the Major Histocompatibility Complex (MHC) is well characterized in the anuran *Xenopus*, this region has not previously been studied in another popular model species, the common frog (*Rana temporaria*). Nor, to date, have there been any studies of MHC in wild amphibian host-pathogen systems. We characterise an MHC class I locus in the common frog, and present primers to amplify both the whole region, and specifically the antigen binding region. As no more than two expressed haplotypes were found in over 400 clones from 66 individuals, it is likely that there is a single class I locus in this species. This finding is consistent with the single class I locus in *Xenopus*, but contrasts with the multiple loci identified in axolotls, providing evidence that the diversification of MHC class I into multiple loci likely occurred after the Caudata/Anura divergence (approximately 350 million years ago) but before the Ranidae/Pipidae divergence (approximately 230 mya). We use this locus to compare wild populations of common frogs that have been infected with a viral pathogen (*Ranavirus*) with those that have no history of infection. We demonstrate that certain MHC supertypes are associated with infection status (even after accounting for shared ancestry), and that the diseased populations have more similar supertype frequencies (lower F_ST_) than the uninfected. These patterns were not seen in a suite of putatively neutral microsatellite loci. We interpret this pattern at the MHC locus to indicate that the disease has imposed selection for particular haplotypes, and hence that common frogs may be adapting to the presence of *Ranavirus*, which currently kills tens of thousands of amphibians in the UK each year.

## Introduction

Many studies searching for the genetic consequences of natural selection have focused their attention on the Major Histocompatibility Complex (MHC) [e.g. 1], [2], [3]. The MHC is a group of genes known to be directly involved in the immune response in vertebrates, and variation at the MHC has been linked to disease resistance and susceptibility (e.g. contrasting allelic frequencies in resistant and susceptible chickens with Marek's disease [4]). In most vertebrates the MHC comprises several loci which fall into three subgroups, class I, II and III [5]. Proteins encoded by the MHC class I and II loci form complexes with antigenic peptides and present these at the cell surface to the T-cell antigen receptors. The class III region encompasses many genes and although some are involved in immunity, they are not directly involved in the presentation of antigens [6]. MHC class I is primarily involved in presenting endogenously synthesised antigens such as viral proteins, whereas MHC class II is mainly involved in presenting exogenous antigens such as bacteria [7]. However, professional antigen presenting cells (e.g. dendritic cells) can load exogenous antigens onto both class I and II molecules, and autophagy can deliver endogenous antigens to the class II pathway [8]. The class I gene family is divided into the classical class Ia genes and the non-classical class Ib genes. Class Ia genes are very polymorphic, with most of the polymorphism occurring within the Peptide Binding Region (PBR) which is the area that recognizes antigens [9]. Class Ib genes have limited or no polymorphism and their function remains largely unknown [9], [10].

Expressed MHC loci typically show very high levels of diversity, which correspond to allelic polymorphisms [11]. High diversity in MHC loci could have a straightforward benefit, by allowing the identification of a larger number of antigens, and so enabling the organism to cope with a greater range of pathogens. It has therefore been proposed that the high diversity at the MHC is maintained by balancing selection, caused by coevolution between hosts and pathogens. One possible form of balancing selection is overdominance (heterozygote advantage), whereby heterozygotes are at an advantage as they can present a broader range of antigens [12]. An alternative form is negative frequency-dependent selection, whereby individuals with a novel rare allele have a selective advantage, since pathogens have not evolved to escape their surveillance [13], [14]. Although overdominance and negative-frequency dependent selection are the most frequently proposed modes of selection at the MHC, there are other possibilities. For example, there is evidence for fluctuating selection in TAP genes (Transporter associated with Antigen Processing) in Danish brown trout [15]. Fluctuating selection occurs when a heterogeneous environment causes selection for different alleles over time and/or space [16]. Fluctuating selection could occur if a pathogen is fast evolving, or if different strains occur in different populations. Directional selection can also occur at the MHC, in which case the spread of an advantageous allele (positive selection) would be expected to lead to a loss of genetic variation. Similarly, selection against disadvantageous alleles (negative or purifying selection) would also be expected to reduce diversity. Directional selection may explain why the two human leukocyte antigen (HLA) types associated with protection from malaria are common in West Africans but rare in other racial groups [17]. Directional selection appears most likely when a single pathogen imposes a substantial proportion of the selection on a particular host species.

In *Xenopus laevis* there is a single class I locus of the class Ia type [18], [19], and there is evidence that this locus is involved in susceptibility to viral infection (*Ranavirus*, family: Iridoviridae) in amphibians. Pre-metamorphic *Xenopus* tadpoles do not express MHC class Ia genes [20] and have been shown (in laboratory experiments) to have much higher mortality rates when exposed to *Ranavirus* than adults which do express these genes [21]. Laboratory studies on *Xenopus* also imply that the MHC genotype may play a role in lethality, with different genotypes conferring differing levels of susceptibility to *Ranavirus* infection [21]. Many wild populations of common frogs (*Rana temporaria*) in the UK have been infected with *Ranavirus*, which causes skin ulceration, systemic haemmorhaging, and can result in mass mortalities [22]. *Ranavirus* is estimated to kill tens of thousands of common frogs in the UK each year [23].

In this study we characterised MHC class I in the common frog and developed primers to amplify exons 2 and 3 which form the antigen binding region (á1 & á2 domains). We use this novel MHC marker to explore selection in wild populations of common frogs with and without a history of *Ranavirus* infection. The aims of this study were (1) to identify whether there are specific MHC supertypes associated with infected (Rv+) and uninfected (Rv−) populations, and thus assess evidence for selection in this system; and (2) to compare MHC diversity with previous information on neutral diversity at microsatellite loci in the same populations.

## Results

**Figure 1:**
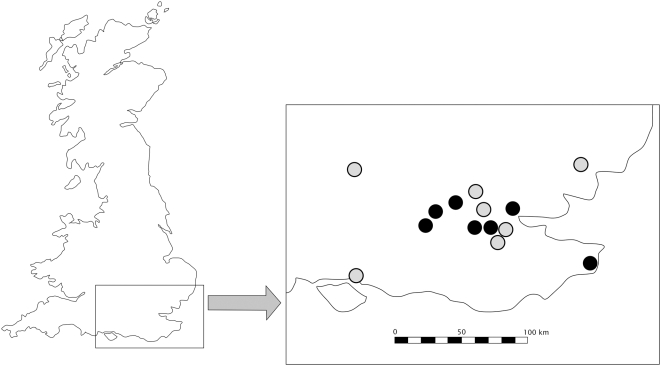
Map Showing the Location of Study Sites in England. Infected (Rv+) populations are indicated by black circles, and uninfected (Rv−) by grey circles.

Full MHC genotypes, based on criteria outlined in the methods section (6+ identical clones for a homozygote, 2+ clones of each allele for a heterozygote), were obtained for 32 Rv− individuals (7 populations, 4–5 individuals per population) and 31 Rv+ individuals (7 populations, 4–5 individuals per population) (see Fig. 1 for sampling locations). Single confirmed alleles were obtained for a further 2 Rv− individuals and one Rv+ individual. The 129 alleles from over 400 clones revealed a maximum of two alleles per individual. The MHC region was highly variable, with 178 out of 543 (32.78%) nucleotides, and 90 out of 181 (49.72%) amino acids, being polymorphic. The mean proportion of polymorphic nucleotides was significantly greater in the Rv− group compared to the Rv+ group (Rv+ = 0.0793, Rv− = 0.0869, p<0.0001). Heterozygosity in Rv+ (0.875) and Rv− (0.936) groups did not differ significantly from expected heterozygosity (Rv+ = 0.965, Rv− = 0.972), and did not differ significantly between groups.

**Figure 2:**
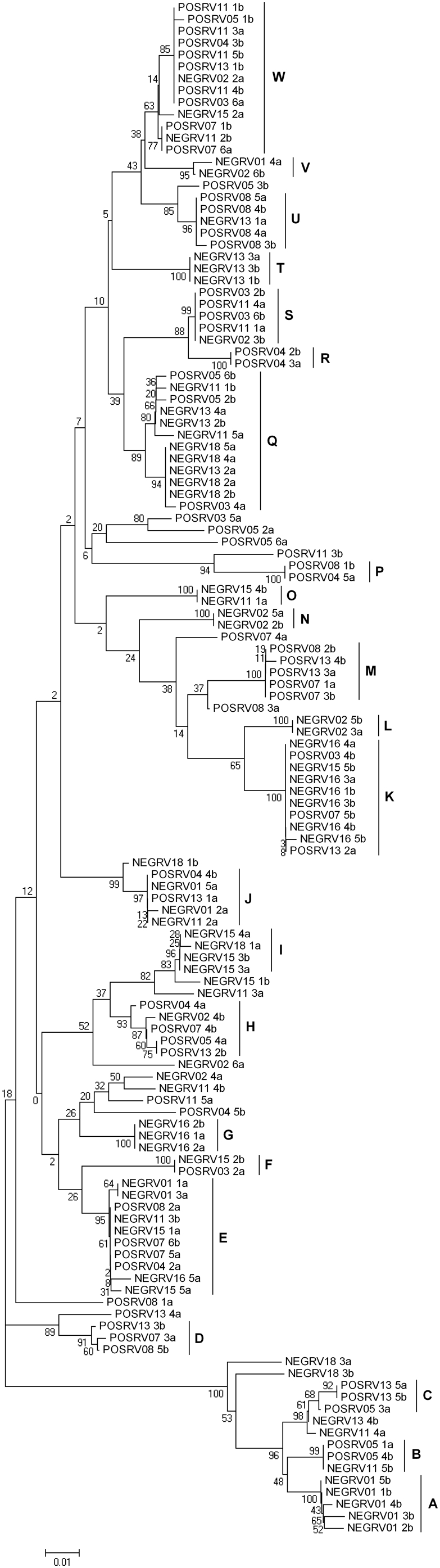
Neighbour-Joining Tree of MHC Sequences with Supertypes Labeled. Individuals are labeled with disease status ‘POS’ (Rv+) or ‘NEG’ (Rv−), population ID, individual ID and allele ID. Bootstrap support values are reported by each node.

**Figure 3:**
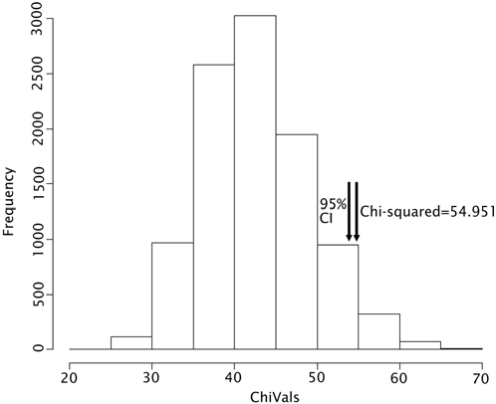
Distribution of Chi-squared Values Generated by Randomizing Disease Status. The chi-squared value obtained from empirical data is marked with an arrow, together with the 95% confidence limit.

Modeltest [24] identified the TrN+I+G model as that which best fitted our data. This model allows for variable base frequencies, equal transversion frequencies and variable transition frequencies, with a proportion of invariable sites (I = 0.349) and a gamma distributed rate variation among sites (G = 0.248) [25]. A neighbour joining tree of the MHC sequences revealed 23 supertypes (Fig. 2). The supertypes differed significantly in frequency between Rv+ and Rv− populations, even after accounting for shared ancestry (χ^2^ = 54.951, p<0.05) (Fig. 3). The comparable test on fourteen microsatellite loci in the same populations only identified one additional locus (RtSB3 [26]) that showed significant (without Bonferroni correction) frequency differences between Rv+ and Rv− populations (χ^2^ = 79.53, p<0.05).

Analysis using Fdist2 [27] showed that the genetic variation among populations (as measured by F_ST_ among Rv+ and Rv− populations combined) was significantly greater at the MHC locus than the average of the microsatellite loci (F_ST_ = 0.150, p<0.03), indicating that this locus may be under some form of selection. Much of this effect was due to variation among the Rv− populations (F_ST_ = 0.214, p<0.008), whereas the Rv+ populations were genetically more similar in their MHC allele frequencies and not significantly more variable than the microsatellites (F_ST_ = 0.066,p = 0.578). As would be expected from the comparison of frequencies in Rv+ and Rv− sites, the microsatellite locus RtSB3 also showed significantly elevated F_ST_ in the pooled populations, as did an additional locus RtSB14 [26] – however neither showed the difference in F_ST_ within Rv+ populations, and only RtSB14 showed this pattern within Rv− populations.

## Discussion

It is apparent from our data that there is a single expressed MHC class I locus in *Rana temporaria*, as we found a maximum of two alleles per individual (n = 66 individuals). *Xenopus* species have also been shown to have a single class I locus [28], in contrast to axolotl [29], indicating that *Rana temporaria* may have a primordial MHC organization like that of *Xenopus*. The Caudata (including axolotls) are thought to have diverged from Anura (including *Rana* and *Xenopus* sp.) over 350 million years ago, whilst Ranidae (*Rana* sp.) and Pipidae (*Xenopus* sp.) diverged approximately 230mya [30]. This implies that the diversification of MHC class I into multiple loci occurred between these two dates.

It has previously been shown that MHC Class I deficient tadpoles (this locus is not expressed until metamorphosis) are more susceptible to *Ranavirus* infection than adults, when exposed to the virus in the laboratory [21], [31]. The authors also noted that a particular *Xenopus* strain showed a high tadpole mortality rate and a slower adult recovery time; suggesting tentatively that the genotype of this strain might infer higher susceptibility, though no sequence information was presented [21]. Our evidence comes from a natural experiment in the field, in which some ponds have been exposed to repeated infection for over a decade, whereas others have escaped disease over the same period. We have shown that certain MHC class I supertypes differ in frequency between infected and uninfected wild populations of common frogs, implying directional selection against the alleles conferring greater susceptibility. This finding indicates that the frogs are adapting to the presence of *Ranavirus* in the wild. Other research has shown that *Xenopus* is capable of mounting a specific immune response to *Ranavirus* under laboratory conditions [32]; it is possible that this is also occurring in the Rv+ populations in our study.

The reduced allelic richness in Rv+ populations might be explained by a bottleneck due to the frog mortality [33]. However, it has previously been shown that there was no comparable reduction in allelic diversity at microsatellite loci in infected populations, comparing these and also 11 further Rv+ and 9 further Rv− populations (Teacher 2009, *PhD thesis*, *University of London*). Hence the reduction in MHC richness appears better explained as another effect of directional selection, which could also produce more uniform allele frequencies (the reduced F_ST_ among the Rv+ populations) if the different infected ponds retained similar resistant genotypes. This finding implies that *Ranavirus* imposes selection for similar genotypes across the geographical range studied.

Microsatellite loci RtSB3 and RtSB14 also showed a higher F_ST_ than expected, although only RtSB3 showed a significant difference between Rv+ and Rv− ponds under the more stringent randomization test. It is possible that these loci may be linked to an adaptive locus (i.e. had their frequencies changed by genetic hitchhiking [34]). It would be interesting to identify candidate genes by chromosome walking or genome mapping.

The microsatellite studies had provided evidence for positive assortative mating in Rv+ populations: elevated homozogosity indicated an excess of mating between related individuals. There is therefore an intriguing possibility that assortative mating for resistant MHC haplotypes may be taking place. Experimental studies of resistance/susceptibility of MHC genotypes under deliberate *Ranavirus* infection, and associated mate choice could prove fruitful for understanding the mechanism fully.

## Materials and Methods

### Ethics Statement

This work was performed under UK Home Office licensing, and ethics committee approval from the Zoological Society of London.

### Sampling

Common frog populations were chosen based on their disease history. Seven populations where chosen which had undergone yearly mortalities caused by *Ranavirus* for approximately 5 generations (10 years) (Rv+ populations), and seven populations were chosen which had no history of *Ranavirus* mortalities (Rv− populations). All populations were located in the South-East of England (Fig. 1) in urban or sub-urban garden ponds. Frogs were captured by hand at each location; each frog was then weighed and held in a clean container at ambient temperature. Frogs below 15 grams were released due to the risks associated with the blood sampling procedures on small animals. Between 5 and 10 frogs of a suitable size were sampled at each location. Each frog was anaesthetised using an aqueous solution of MS222 (Pharmaq Ltd, UK) buffered with NaHCO_3_ to the pH of the water where the frog was collected (or at pH 7 if collected on land). Each frog was then bled via cardiocentesis using 0.5 ml heparinised insulin syringes, and blood samples of 150 µl maximum volume were collected. The frogs were then rinsed in fresh water until conscious, and placed in their original container until all signs of anaesthetic exposure had passed for at least 10 minutes. They were then released back into the wild at the location of capture, taking care to ensure that day-time release did not expose the animals to any additional hazards. Blood samples were centrifuged to pellet the red blood cells, and the pellets were stored in RNALater (Ambion, UK).

### Molecular techniques

Total RNA was extracted from blood pellets using the SV 96 Total RNA Isolation System (Promega, UK) which includes DNase treatment to remove genomic DNA (gDNA). The standard protocol for this kit was altered due to the low volumes of blood available; in the first lysis step 40 µl blood was added to 300 µl RNA Lysis Buffer. RNA concentrations of approximately 0.03 µg/ml were obtained and cDNA synthesis was performed using the First Strand cDNA Synthesis Kit (GE Healthcare/Amersham).

Published MHC class I mRNA sequences from cDNA clones in *Rana pipiens* (GenBank accession numbers AF185587 and AF185588) were aligned using ClustalW, and Primer3 [35] was used to design primers to amplify the whole MHC class I region (approximately 900 bp): Rpip9F 5′-ttccgacagtcacactctgc-3′ and RpipR 5′-ggtggtcttgtagccttctcc-3′. Once *Rana temporaria* sequences were obtained from these primers, a species-specific reverse primer (RpipR2 5′-tgaaacccgtacaccagaca-3′) was designed to be used with Rpip9F to amplify only the antigen binding region (exons 2 and 3 corresponding to the á1 & á2 domains, approximately 650 bp). The 650 base-pair region was amplified from cDNA using the Polymerase Chain Reaction (PCR) with primers Rpip9F and RpipR2. The reagents were: 16 µl Taq PCR Master Mix (Qiagen, UK), 4 µl cDNA, 200 pmol of each primer (2 µl), and 16 µl RNAse-free water. The samples were denatured at 95°C for 15 minutes followed by 25 cycles of 94°C for 30 sec, 60°C for 1 min 30 sec, and 71°C for 2 minutes, followed by a final elongation step of 71°C for 10 min to complete fragment extension. The PCR products were run on 1.2% agarose gels with 5 µl loading buffer and a 100 base-pair ladder (Microzone Ltd, UK). The 650 base pair bands were cut from the gels and DNA extracted using the QIAquick Gel Extraction Kit (Qiagen, UK). One sample of gDNA was run with each cDNA PCR plate; gDNA samples yield a 950 base pair band which is not seen in cDNA amplifications, thus confirming that cDNA samples were not contaminated with gDNA.

Ligation and transformation of amplified cDNA products were performed the same day using the Qiagen PCR Cloning Plus Kit (Qiagen, UK). For each individual, 75 µl of transformed cells were spread onto an LB agar plate containing ampicillin, IPTG and X-gal according to the cloning kit instructions. Plates were incubated overnight at 37°C. Six clonal sequences were required per individual to reduce the chances of missing one allele from a heterozygote to below p = 0.05. Seven colonies were picked per individual as colonies did not always contain the insert. The colonies were grown overnight at 37°C, shaking at 225 rpm, in individual falcon tubes containing 600 µl LB broth with 30 µg ampicillin. Plasmid purifications were then prepared from the colonies using the Zyppy Plasmid Miniprep Kit (Zymo Research, USA). Purified plasmid DNA (30–50 µg/ml) was used for sequencing with standard M13 primers by Cogenics (Essex, UK).

### Analysis

Alleles were confirmed when at least two clones showed identical sequences. Homozygotes were confirmed on the basis of at least 6 identical sequences; when 6 clones are picked, there is a probability of 0.5^5^ = 0.03125 that there is a second allele which has not been picked by chance. Every confirmed allele was recorded in the data set. Nucleotide sequences (GenBank accession numbers FJ385575–FJ385703) were aligned using Sequencher v.4. (Gene Codes Corporation), and confirmed as belonging to the MHC Class I conserved domain using BLAST searches (National Center for Biotechnology Information). We used the antigen binding region (comprising of the α1 and α2 domains) for all analyses; this region which was identified by amino acid alignment with Xenopus [36] and Atlantic salmon [1] sequences.

Observed heterozygosity was calculated within Rv+ and Rv− groups by dividing the number of observed heterozygotes by the total number of individuals with both alleles typed. Expected heterozygosity was calculated as *H_E_* = 1−Σ *p_i_* ^2^ where *p*_i_ is the frequency of the *i* ^th^ allele. The observed number of heterozygotes was compared with Hardy-Weinberg expectations [37] within Rv+ and Rv− groups using Fisher's exact test, and between groups using a Chi-squared test; both tests were implemented in R v.2.4.0. The allelic richness was characterized by the mean number of substitutions per base as calculated by MEGA [38], and compared between Rv+ and Rv− groups using a two-tailed t-test. Modeltest [24] was used to test 56 possible DNA substitution model parameters, and identify the best fit model for the data. A neighbour-joining tree was produced using the Tamura-Nei model of nucleotide substitution [25], with 100 bootstrap replicates. MHC supertypes were identified on the basis of branch length; clusters of individuals (n>1) which were separated from other individuals by a branch length of ≤0.007 (an arbitrarily chosen value, after visual inspection of data) were categorized as a unique supertype (Fig. 2). A Chi-squared test was used to compare MHC supertype frequencies between Rv+ and Rv− populations; however the individuals within each category are not strictly independent, as inhabitants of the same pond are likely to share common ancestry. To overcome this issue, we constructed a randomization test (performed in R v.2.4.0). The Chi-squared test was repeated (for 10,000 iterations) after reallocating disease status at random to ponds; the source code is included in Supporting Information S1. The Chi-squared value from the real data was then compared with the randomized distribution to establish the significance level. For comparison, this method was then repeated with data from 14 putatively neutral microsatellite loci (Teacher 2009, *PhD thesis*, *University of London*), using data for the same populations as used for the MHC analyses (Rv+ = 77 individuals, mean per population = 11; Rv− = 61 individuals, mean per population = 9) to investigate whether the Rv+ and Rv− populations were, by chance, more genetically distinct than average. We used FDist2 [27] implemented in Lositan [39] to further assess the effect of any selection on the variation between populations at the MHC or microsatellite loci. The program compares the F_ST_ of marker loci with simulated expected values (conditional on their heterozygosity), and identifies outliers. This method was applied to Rv+ and Rv− populations individually and combined, using data at the MHC locus, together with data for the same populations from the 14 microsatellite loci to provide information on neutral expectations.

## Supporting Information

Supporting Information S1 (0.03 MB DOC)
