# Supplementary material for: Evidence for Directional Selection at a Novel Major Histocompatibility Class I Marker in Wild Common Frogs (Rana temporaria) Exposed to a Viral Pathogen (Ranavirus)
Source: PLoS One. 2009 Feb 25;4(2):e4616. doi: 10.1371/journal.pone.0004616 (PMC2643007; doi:10.1371/journal.pone.0004616)
Supplement: Supporting Information S1 — (0.03 MB DOC) [file pone.0004616.s001.doc]

**Supporting information S1: R code for randomization of disease status and generation of Chi-squared distribution**

dat<-read.table(header=T,'MHCcomb.txt')

attach(dat)

Pop<-as.factor(Pop)

Nalleles<-nlevels(Allele)

D<-matrix(nrow=Nalleles,ncol=2)

for (r in 1:Nalleles) D[r,1]=sum((Allele==levels(Allele)[r]) & (Disease=="p"))

for (r in 1:Nalleles) D[r,2]=sum((Allele==levels(Allele)[r]) & !(Disease=="p"))

chisq.test(D,simulate.p.value=T,B=10000)

numiters<-10000

ChiVals<-vector(length=numiters)

for (iter in 1:numiters){

tempD<-is.element(Pop,sample(levels(Pop),7))

for (r in 1:Nalleles) D[r,1]=sum((Allele==levels(Allele)[r]) & tempD)

for (r in 1:Nalleles) D[r,2]=sum((Allele==levels(Allele)[r]) & !tempD)

ChiVals[iter]<-chisq.test(D)$statistic }

quantile(ChiVals,c(0.5,0.95,0.99))

hist(ChiVals)
